# Spectrum of MR imaging findings of sinonasal mucormycosis in post COVID-19 patients

**DOI:** 10.1259/bjr.20210648

**Published:** 2021-10-07

**Authors:** Sushen Kumar Kondapavuluri, Varun Kumar reddy Anchala, Shohyle Bandlapalli, Rajani Gorantla, Ankamma Rao Danaboyina, Benod Kumar Kondapavuluri, Satyanarayana Mandalapu

**Affiliations:** 1 NRI Academy of Medical Sciences, Guntur, India

## Abstract

During rapid spread of coronavirus disease (COVID-19) globally, ever since WHO declared COVID-19 as pandemic, there have been various patterns of disease in terms of diagnosis, management and complications. Secondary infections are reportedly common in hospitalized and severely ill COVID-19 patients among which fungal being 10 times more common. Mucormycosis is amongst the most lethal form of Zygormycosis occurring in post COVID-19 patients. A varied patterns of disease involvement and spectrum of imaging features are observed in patients with mucormycosis in post COVID-19 patients. MRI has better efficacy than CT in detecting early invasion of mucormycosis. The goal of this review is to familiarize radiologists about the MR imaging spectrum of mucormycosis in post COVID-19 patients with potential diagnostic pitfalls in CT.

**Advances in knowledge:**

Radiological findings of mucormycosis in post COVID-19 patients show varied patterns of disease involvement and spectrum of imaging features. One should not solely rely on CT imaging to detect the extent of disease. MRI helps in early and accurate detection of invasion into adjacent structures and so helpful in early intervention.

## Introduction

**Figure 1.**
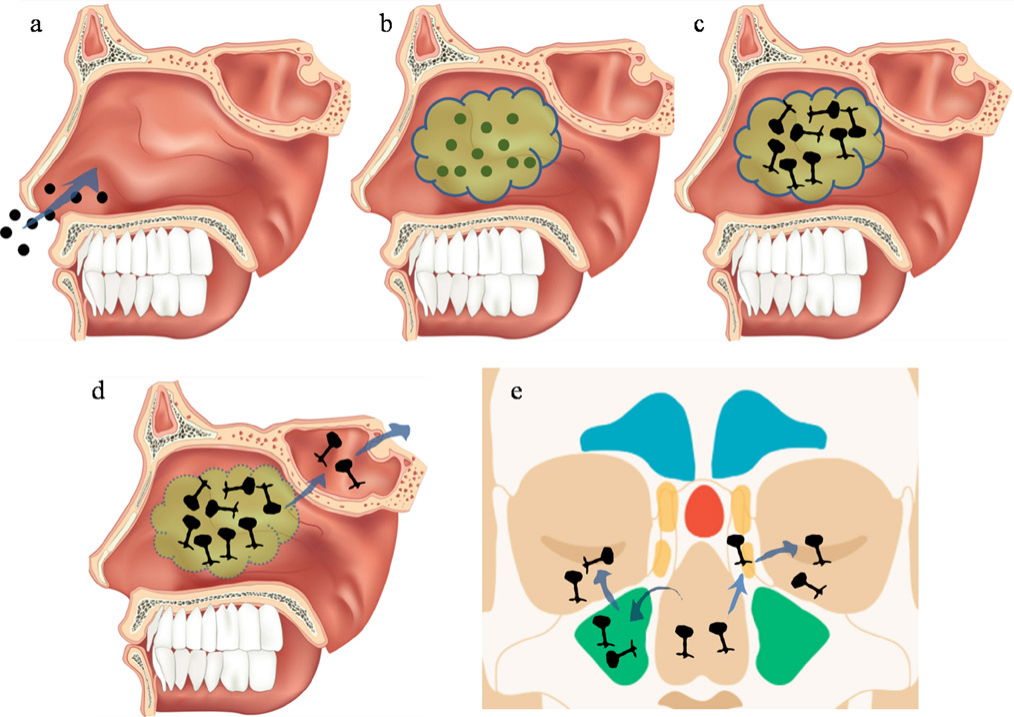
**(a**) Inhalation of fungal spores into the nasal cavity. (**b**) Inoculation of fungal spores. (**c**) Due to metabolic hypoxic conditions inoculated spores undergoes germination. (**d**) Mucorales invade the bone with extension into sphenoid sinus and further intracranially. (**e**) Extension of the disease into maxillary and ethmoid sinuses can lead to orbital involvement.

During rapid spread of coronavirus disease (COVID-19) globally, ever since WHO declared COVID-19 as pandemic, there have been various patterns of disease in terms of diagnosis, management and complications.^1^ Despite strenuous efforts, there is no definitive treatment of the disease till date. In India, after the first wave of cases in 2020, there is a rapid surge in cases of COVID-19 since march 2021,^2^ which is more deadly with almost equal rise in cases of secondary infections. Secondary infections are reportedly common in hospitalized and severely ill COVID-19 patients among which fungal being 10 times more common. Mucormycosis is amongst the most lethal form of Zygormycosis occurring in post COVID-19 patients.^3,4^ As the nature of the disease is still inconclusive, it cannot be confirmed if it is a complication of the disease or its management. Mucormycosis commences with either reactivation of nasal colonization or nasal inoculation of spores which germinates and then spread rapidly through various routes (Figure 1).

Clinically, fungal sinusitis can be present with atypical signs and symptoms similar to complicated sinusitis such as nasal blockade and headache. Patients with intraorbital extension present with ophthalmoplegia, facialedema and proptosis and various neurological signs and symptoms if intracranial extension in present. Without early diagnosis and intervention, there may be rapid progression of the disease, with reported mortality rated from intraorbital and intracranial complications of 50–80%.^5^

Recently, a spike in the incidence of fungal infection has been observed in post Covid-19 patients, with more cases being diagnosed much more frequently. MRI is superior than CT owing to its increased slice orientation as well as superior anatomic and pathologic resolution. The ability to depict cross-sectional anatomy and pathology with better tissue characterization and even without administering intravenous gadolinium-based contrast agent is a distinct advantage of MRI over CT scanning. Contrast administration is useful in differentiation viable from a dead necrotic tissue in sinusitis with an added advantage of differentiating abscess from phlegmon in orbit and intracranial extensions. MRI has a highest accuracy in the detection of cavernous sinus involvement and perineural spread, which are uncommon but serious complications of fungus sinusitis. The goal of this review is to familiarize radiologists about the MR imaging spectrum of mucormycosis in post COVID-19 patients with potential diagnostic pitfalls in CT. The study was approved by ethics committee of our hospital.

### MRI protocol

All patients underwent MRI scan on 1.5 Telsa MR Scanner (GE) using a 16 channel neurovascular coil (NV) −16 coil. Multiplanar, multiecho MRI of paranasal sinuses with orbits and brain sections were performed with the set protocol (Table 1).

**Table 1.** MRI protocol

Plane	Sequences
Axial	T1,T2,T2FS, DWI, ADC, SWI
Coronal	T2,T2FS
Sagittal	T2FS
3D Bravo	T1 FS Pre-contrast and T1FS Post-contrast

Spectrum of mucormycosis in post COVID-19 patients based on involvement of various anatomical structures (Table 2).

**Table 2.** Spectrum of involvement of mucormycosis in post COVID-19 patients

Site	Pattern of involvement
Sinuses	Maxillary and ethmoid sinuses are most commonly involved
Bones	Black turbinate sign Isolated marrow involvement (Rare)
Soft tissues	Pterygomaxillary fissure Premaxillary soft tissues
Orbital involvement	Pre and post septal cellulitis “guitar pick” sign Optic nerve ischemia
Intracranial	Cavernous sinus involvement Perineural invasion Angioinvasion Brain parenchymal involvement Meningeal involvement

### Sinus involvement

**Figure 2.**
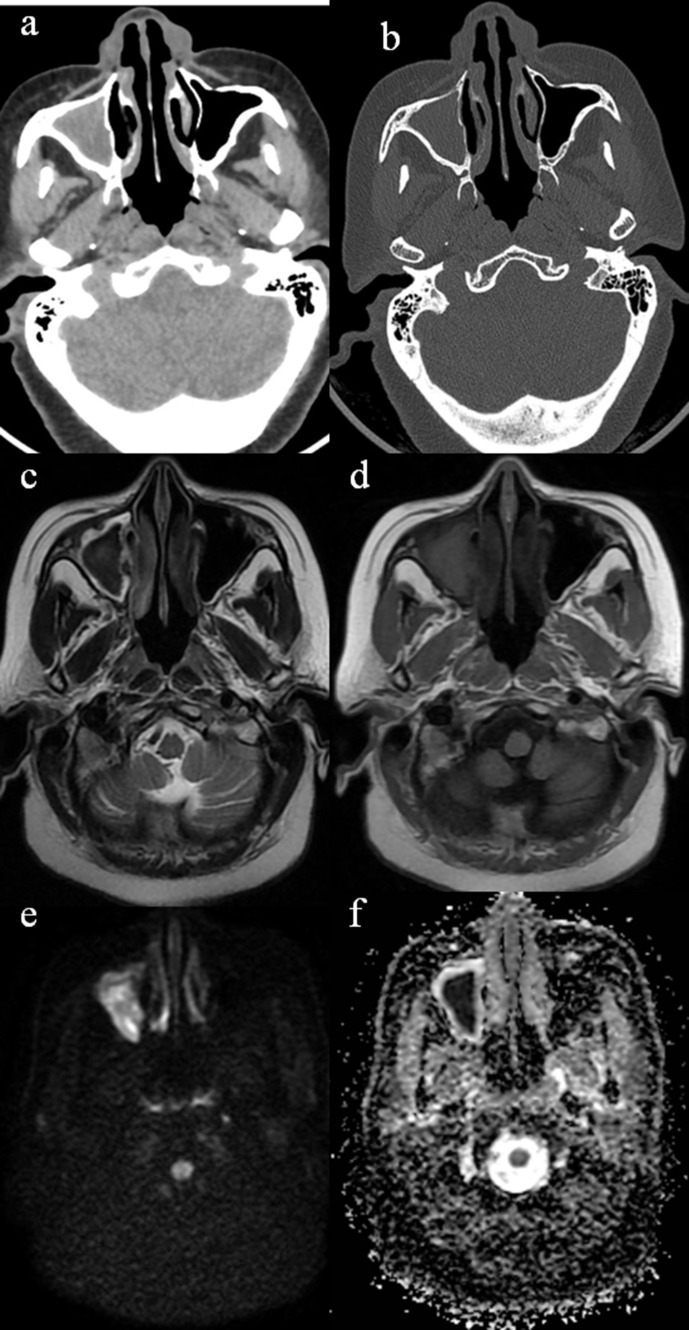
CT Axial soft tissue (**a**) and bone window images of mucormycosis shows complete opacification of right maxillary sinus (**a**) without any bone erosions (b). Axial MR images of same patient. Lesion shows hypointense signal on T2W images (**c**) and hyperintense signal on T1W images (**d**). Diffusion sequence images (e, b 1000; f, ADC map) show restricted diffusivity with low ADC value.

Among all the sinuses maxillary and ethmoid sinuses are most commonly involved.^6^ On CT, mucormycosis typically appears hyperdensity owing to its intralesional metal-dense spots corresponding to fungal waste products. MR imaging shows variable signal intensities on *T*_1_-weighted and *T*_2_-weighted images; however, fungal elements themselves cause low signal intensities on *T*_2_-weighted imaging. DWI sequence may aid in diagnosis of fungal sinusitis, which shows increased signal intensity of affected sinus with low ADC values. Absence of hyperdensity on CT does not rule out mucormycosis as MRI is the preferred modality of choice, which shows changes at the earliest (Figure 2).

### Bone involvement

**Figure 3.**
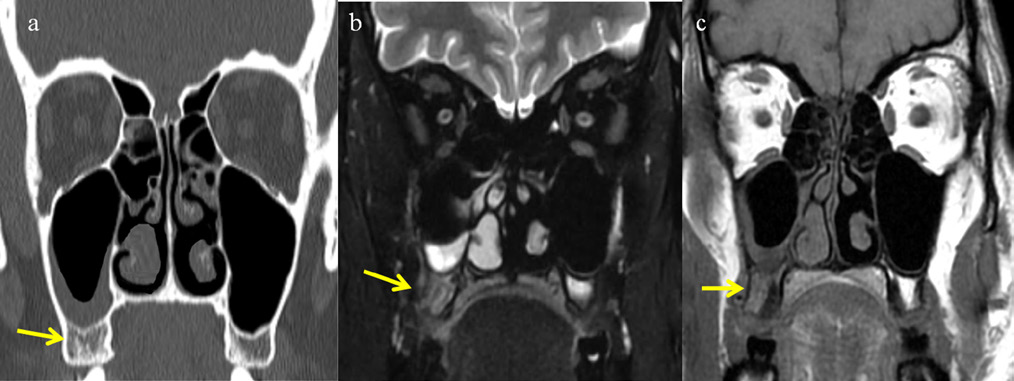
Coronal CT and MRI images of early mucormycosis showing bone marrow infiltration. (**a**) Coronal CT (Bone window) showing mucosal thickening in right maxillary sinus. Bone involvement is not clearly appreciated. Coronal T2FS(b) and coronal T1W (**c**) images showing abnormal hyperintensity (arrow) in right alveolar process of maxilla (b) and replacement of normal fatty marrow (arrow) compared to the normal opposite side (**c**).

**Figure 4.**
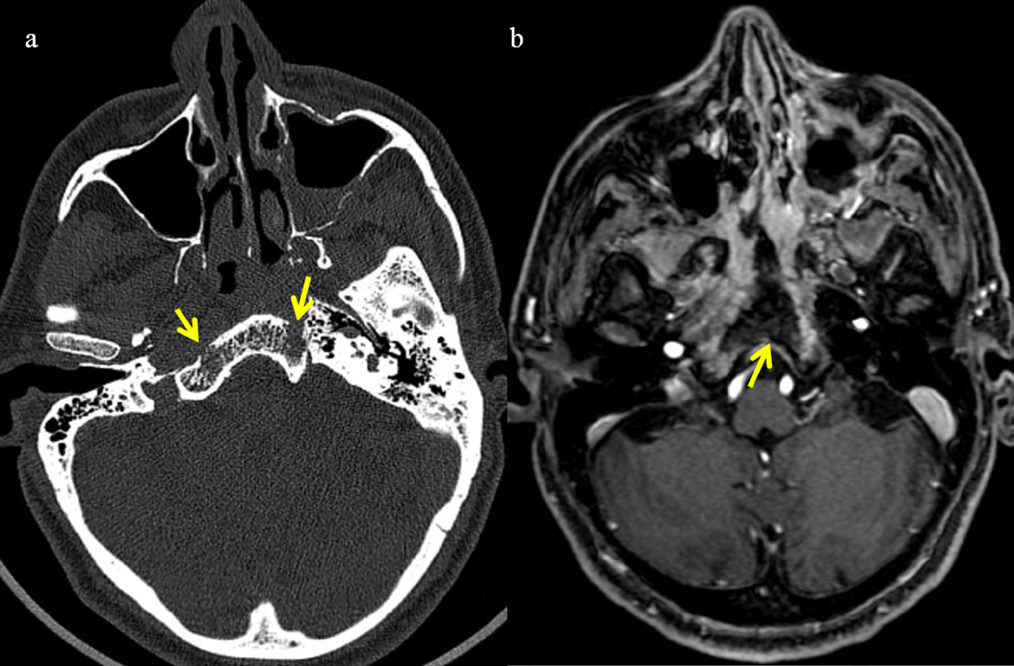
Axial bone window section of CT(a) and post-contrast axial T1FS MRI (b) showing bone invasion. (**a**) Permeative destruction noted involving the clivus (arrow). (**b**) Non-enhancing areas involving the clivus indicating necrosis (arrow).

**Figure 5.**
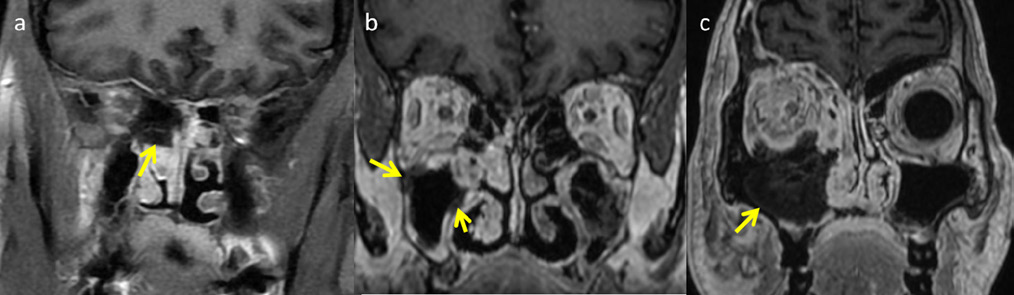
Coronal post-contrast T1W images of mucormycosis in three different patients showing black turbinate sign. (**a**) Non-enhancing lamina papyracea, superior turbinate and superior portion of middle tubinate (arrow). (**b**) Multifocal areas of non-enhancement within the right maxillary sinus (arrow). (**c**) Diffuse non-enhancing areas involving maxillary sinus and floor of orbit on right side (arrow).

Most common imaging features are complete opacification with expansion, erosion, or remodeling and thinning of the sinuses. Paranasal sinus walls may show mottled air foci with irregular bone destruction.^7^ Marrow invasion can be seen in cases of mucormycosis even before erosions in the bone (Figure 3). Bony erosions appear as permeative destruction involving the sinus walls and the contiguous bony structures (Figure 4). Mycotic microvascular involvement of nasal mucosa leads to infarction of surrounding tissue, which is non-enhancing on post-contrast *T*_1_-weighted images. Infarcted tissue shows diffusion restriction on DWI images. This devitalized and necrotic sinonasal mucosa with complete non-enhancement on MR imaging represents “the black turbinate” sign (Figure 5). This represents earliest imaging finding of nasal mucomycosis on MR imaging.^8^

### Soft tissue involvement

**Figure 6.**
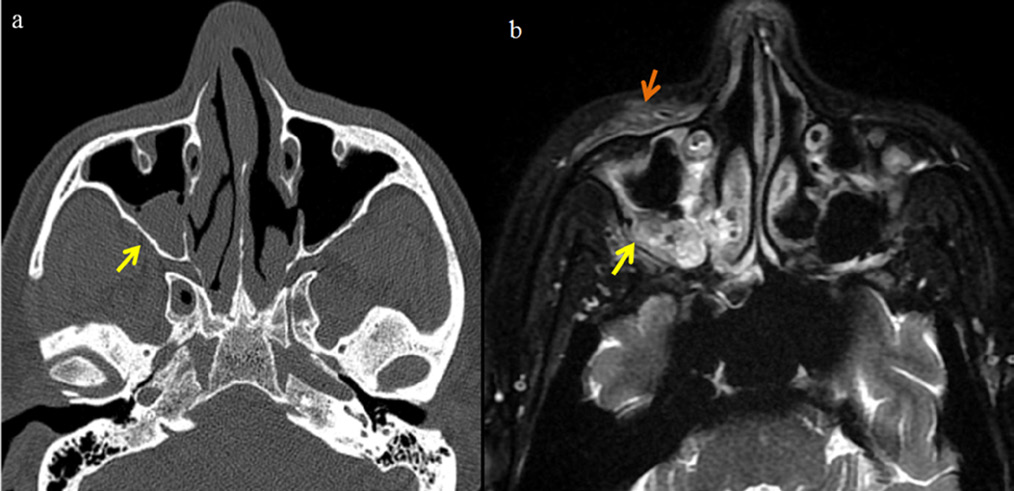
Axial CT bone window image (**a**) shows mucosal thickening in right maxillary sinus without any bone erosions (arrow). T2FS sequence in axial plane showing mucormycosis with soft tissue infiltration. Hyperintensity noted in right premaxillary soft tissues (orange arrow) and pterygopalatine fossa (yellow arrow).

Infiltration of periantral soft tissue is seen as a common imaging finding. Loss of periantral fat is earliest sign of soft tissue involvement. Extension of mucormycosis beyond the sinus can be seen without any bone destruction as it tends to spread along vascular channels and nerves^6^^.^ Spread of mucormycosis from nasal cavity along either posterior nasal nerves or the sphenopalatine artery justifies the involvement of sphenopalatine foremen and ipsilateral pterygopalatine fossa (Figure 6).

### Orbital involvement

**Figure 7.**
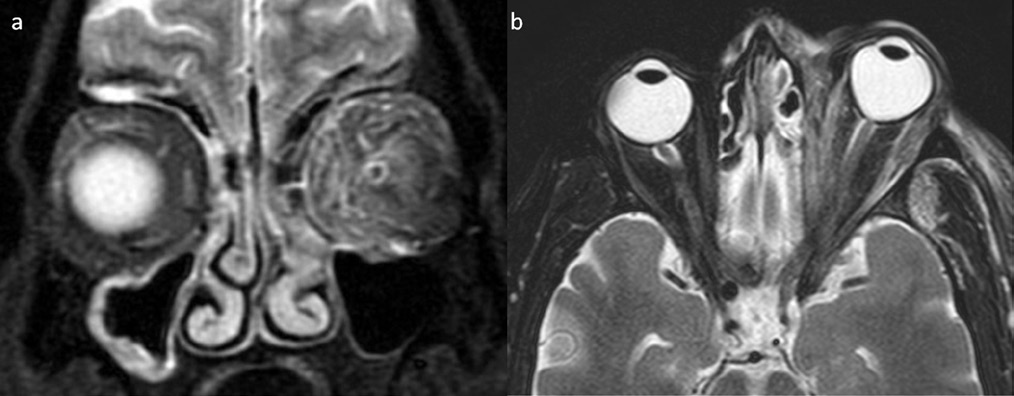
Coronal (**a**) and axial T2FS (**b**) images of mucormycosis showing orbital invasion. (**a**) Hyperintensities noted involving the left orbit indicating orbital cellulitis. (**b**) Proptosis of left eye with inflammatory changes in left orbit.

**Figure 8.**
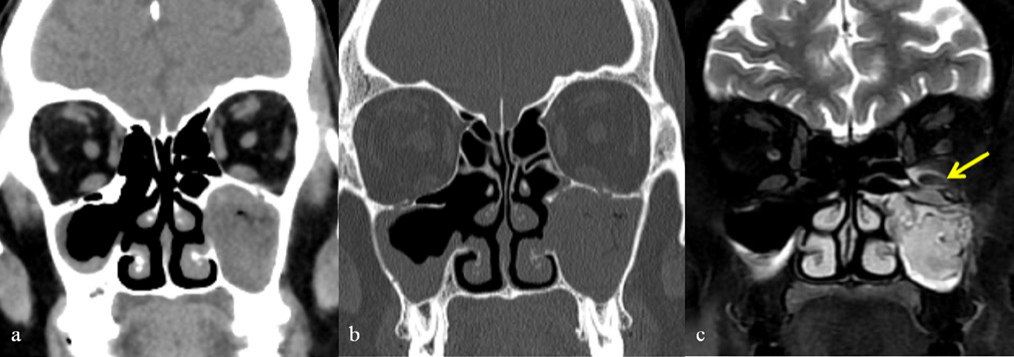
Coronal CT (soft tissue and bone window) and MRI image of mucormycosis. (**a**) and (b) showing complete opacification of left maxillary sinus. No orbital fat stranding detected. Coronal T2FS MR image (**c**) showing inflammatory changes around inferior rectus (yellow arrow) indicating orbital cellulitis.

**Figure 9.**
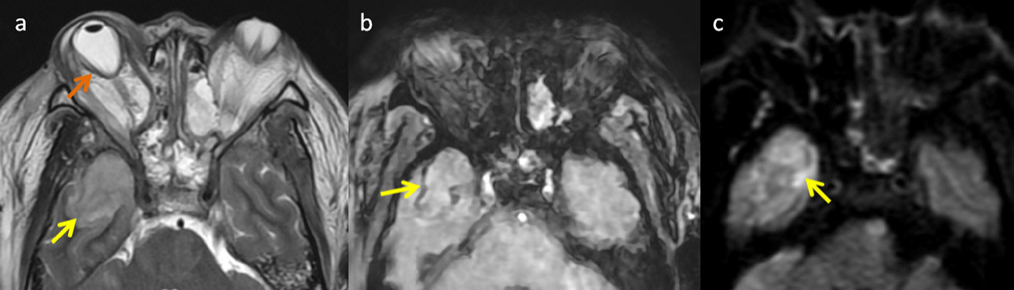
Axial MR images of mucomycosis with cerebritis. (**a**) Abnormal T2 hyperintense involving anterior pole of right temporal lobe (yellow arrow), orbital cellulitis with proptosis and resultant posterior globe tenting (“Guitar pick” sign) (orange arrow). (**b**) Rim of blooming shows in SWI images indicating fibrocollagenous capsule formation. (**c**) Restricted diffusivity on DWI images (arrow).

**Figure 10.**
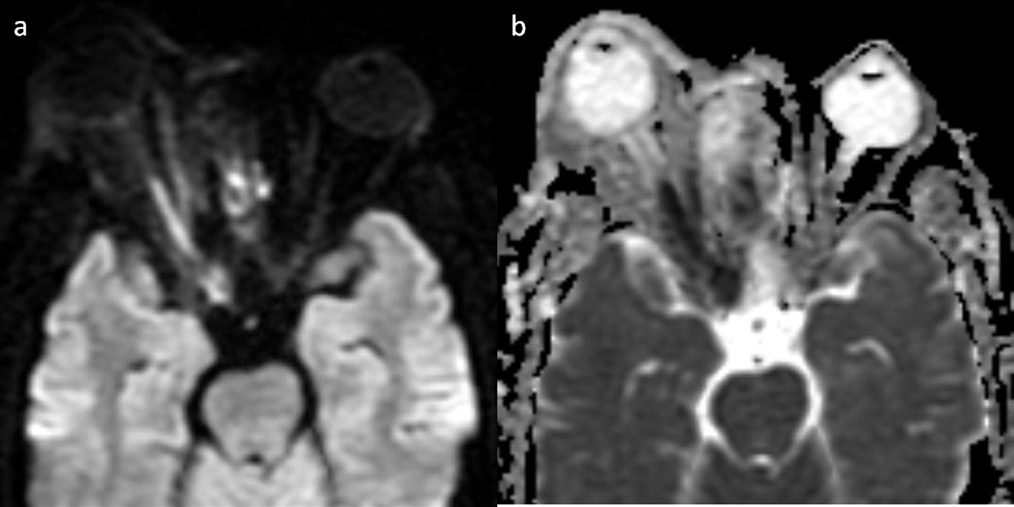
Axial DWI (**a**) and ADC (**b**) images showing optic nerve infarction.

Neuro-ophthalmic involvement of fungal sinusitis develop due to progressive fungal invasion. Close proximity of two structures, thin lamina papyracea and valveless ethmoidal veins influence the spread of mucormycosis from sinuses to orbital fossa. Spreading of disease into orbit and cavernous sinus can also occur due to complex network of veins directly draining from nasal cavity and paranasal sinuses.^9^ Both pre- and postseptal cellulitis can be seen in patients with mucormycosis. On imaging, preseptal cellulitis is limited to the soft tissue anterior to the orbital septum. In contrast, postseptal cellulitis involves the contents of orbit putting optic nerve at risk. Radiologically, postseptal cellulitis typically shows diffuse soft tissue stranding posterior to orbital septum and varied degree of proptosis (Figure 7). One cannot rely solely on CT imaging to rule out orbital extension and should consider MRI in clinically suspected cases of orbital involvement (Figure 8). In cases of severe proptosis, posterior globe shows tenting demonstrating the “guitar pick” sign (Figure 9).^10^ DWI is helpful in detecting optic nerve infarction (Figure 10) and plays a crucial role in differentiating orbital cellulitis from orbital abscess as it is the critical finding, which can alter management from medical to surgical.

### Intracranial involvement

#### Cavernous sinus involvement

**Figure 11.**
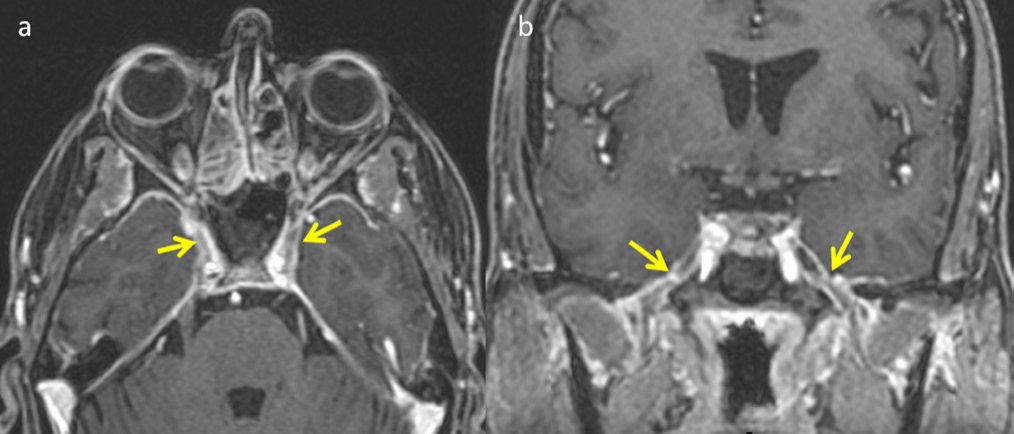
Axial (**a**) and coronal (**b**) postcontrast T1W images showing invasion of cavernous sinus with perineural extension. (**a**) Abnormal enhancement along the bilateral lateral walls of cavernous sinus(arrows). (**b**) Thick enhancing sheet noted extending along mandibular division of trigeminal nerve bilaterally (arrows).

Involvement of orbit is an alarming imaging feature to look for involvement of cavernous sinus. Enhancing soft tissue in orbital apex and cavernous sinus indicates cavernous sinus involvement on imaging. In addition, adjacent ethmoid sinus involvement with bulky and lateral displacement of medial rectus can be seen.^11^ Changes in signal intensity, size and coutour of carvenous sinus and increased dural enhancement along the lateral border of cavernous sinus (Figure 11) indicate cavernous sinus thrombophlebitis.

### Perineural invasion

Perineural invasion is another possible mechanism of central nervous system extension of mucormycosis. Perineural invasion was considered unusual but contrast-enhanced MRI studies have documented perineural invasion via the trigeminal nerve.^12^ Nerve microenvironment and neurotropic factors secreted in a gradient along nerve may play a key role in the pathogenesis of perineural invasion.^13^ On MRI, perineural spread appears as a thick sheet of enhancing tissue along the involved cranial nerve or its branch (Figure 11) in addition to loss of normal fat pad adjacent to a foramen.

### Angioinvasion

**Figure 12.**
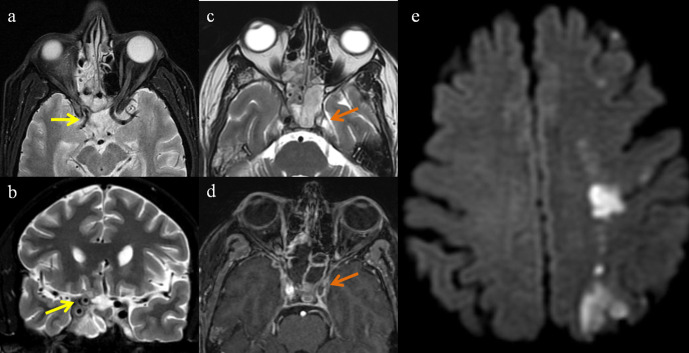
Axial (**a**) and coronal (**b**) T2FS images of mucormycosis showing vessel wall irregularity and thickening (yellow arrow) indicating vasculitis. T2 Axial (**c**), postcontrast axial T1FS (**d**) and DWI (**e**) images of another patient with mucormycosis showing angioinvasion. (**c**) T2W image showing ethmoid and sphenoid sinusitis with loss of flow void in left ICA (arrow).(**d**) Postcontrast T1W image shows no enhancement of left ICA (arrow). (**e**) DWI image shows superficial and deep watershed territory infarcts in left cerebral hemisphere.

Apart from perineural invasion, extensive involvement of vessels with resultant thrombosis and tissue necrosis is the pathological hallmark feature of mucormycosis.^14^ Presence of free iron in plasma and tissues is believed to be crucial for the pathogenesis of vascular invasion. Mucormycosis may show diffuse arterial wall involvement leading to vasculitis or may directly invade into the vessel forming a mucorthrombus (Figure 12).

### Brain parenchymal involvement

**Figure 13.**
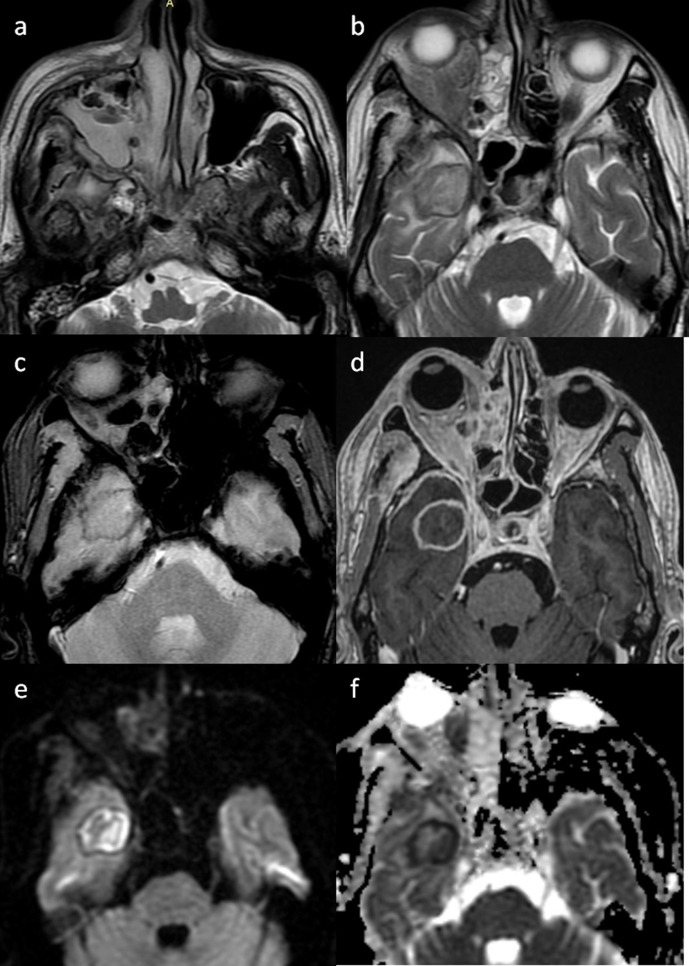
Axial MR images of mucormycosis with intraparenchymal abscess formation. Sinusitis (**a**) with peripherally hypointense lesion on T2W image (b) showing continuous rim of blooming on SWI (c) and showing rim enhancement on postcontrast T1W image (d). Diffusion sequence images (e, b1000; f, ADC map) show diffusion restriction in the periphery and intracavitary projections with low ADC values.

Furthermore devastating complication of mucormycosis is parenchymal involvement, which occurs by invasion through superior orbital fissure, cribriform plate, angioinvasion and perineural route.^15^ As consequence of hematogeneous, spread of mucormycosis causing vasculitis and inflammation of the meninges leads to meningitis. Involvement of brain parenchyma is suggestive of cerebritis and is the precursor of abscess formation (Figure 9). Untreated cases of cerebritis can lead to cerebral abscess formation. DWI sequence is more specific for fungal abscess, which shows restricting wall and intracavitary projections while sparing the core of the lesion (Figure 13).^16^ Characteristic feature of fungal infections on MR spectroscopy is the presence of disaccharide trehalosepeak at 3.6 ppm^16^

## Conclusion

It is still inconclusive about the cause and origin of mucormycosis in post COVID-19 patients. As there is high mortality in patients with complications of mucormycosis, imaging plays a vital role in assessing the involvement of paranasal sinuses, extent of orbital involvement as well as intracranial spread. MRI has high efficacy in early identification of disease process and extent of disease compared to CT. Recognition of potential diagnostic pitfalls is important when interpreting CT studies of mucormycosis.
